# Machine Learning Prediction of Mycobacterial Cell Wall Permeability of Drugs and Drug-like Compounds

**DOI:** 10.3390/molecules28020633

**Published:** 2023-01-07

**Authors:** Eugene V. Radchenko, Grigory V. Antonyan, Stanislav K. Ignatov, Vladimir A. Palyulin

**Affiliations:** 1 Department of Chemistry, Lomonosov Moscow State University, 119991 Moscow, Russia; 2 Department of Chemistry, Lobachevsky State University of Nizhny Novgorod, 603022 Nizhny Novgorod, Russia

**Keywords:** *Mycobacterium tuberculosis*, tuberculosis, resistance, cell wall, permeability, penetration, machine learning, neural networks, fragmental descriptors

## Abstract

The cell wall of *Mycobacterium tuberculosis* and related organisms has a very complex and unusual organization that makes it much less permeable to nutrients and antibiotics, leading to the low activity of many potential antimycobacterial drugs against whole-cell mycobacteria compared to their isolated molecular biotargets. The ability to predict and optimize the cell wall permeability could greatly enhance the development of novel antitubercular agents. Using an extensive structure–permeability dataset for organic compounds derived from published experimental big data (5371 compounds including 2671 penetrating and 2700 non-penetrating compounds), we have created a predictive classification model based on fragmental descriptors and an artificial neural network of a novel architecture that provides better accuracy (cross-validated balanced accuracy 0.768, sensitivity 0.768, specificity 0.769, area under ROC curve 0.911) and applicability domain compared with the previously published results.

## 1. Introduction

Tuberculosis (TB) is a serious infectious disease caused by pathogenic *Mycobacterium tuberculosis* mycobacteria (or, in some cases, by a number of related mycobacteria species belonging to the *Mycobacterium tuberculosis* complex) [1,2]. This is a chronic bacterial infection characterized by the development of cell-mediated hypersensitivity and the formation of granulomas in the affected tissues. The disease is usually localized in the respiratory organs, but other organs may be involved in the process. Tuberculosis exhibits a variety of clinical and pathomorphological manifestations, as well as broad abilities for adaptation to changing environmental conditions and the characteristics of the host organism [3].

According to the World Health Organization (WHO), tuberculosis is one of the most widespread and socially significant infections: every year, despite being a preventable and curable disease, about 10.6 million people develop tuberculosis and 1.6 million people die worldwide, making it the leading cause of death from a single infectious agent [4]. The major problem in the treatment is the mycobacterial resistance to antibiotics. Multidrug-resistant (MDR) mycobacteria are resistant to treatment with two first-line anti-TB medications, isoniazid and rifampicin, whereas the forms that are also resistant to second-line medications are called extensively drug-resistant (XDR) [5,6,7,8].

*Mycobacterium tuberculosis* is a rather complex organism containing a broad variety of targets that can be affected by drug compounds [9,10,11]. The established antimycobacterial targets include specific processes of the cell wall biosynthesis [12,13,14,15], protein synthesis [16], DNA replication and repair [17,18,19], DNA transcription [20], bioenergetic metabolism [21,22,23], and other metabolic pathways [24,25]. In addition, massive and fruitful efforts have been directed in recent years at the identification and exploitation of various emerging and potential targets as the basis for the development of novel antitubercular drugs [26,27,28,29,30,31,32,33], especially with a focus on overcoming the drug resistance [34,35,36]. Besides the enzyme targets, the important roles of mycobacterial membranes [37] and transporters [38,39] as drug targets has been recognized, as well as the opportunities offered by the multi-target approaches [40] and host-directed therapies [35,41]. Promising studies of novel preventive and therapeutic TB vaccines [42,43,44] and nanocarrier-based approaches for the efficient and targeted delivery of anti-TB drugs and vaccines [45,46] are also ongoing.

Nevertheless, the anti-tubercular drug discovery and development projects face many complications that result in high attrition rates, leaving clinical needs unmet [47,48,49]. In particular, the target-based approaches using biochemical screening assays and/or in silico models [50] to identify and optimize inhibitors have so far failed to produce any clinical drug candidates, primarily due to their lack of whole-cell activity [30,48]. It is commonly accepted that one of the key causes for this is the extremely low permeability of the mycobacterial cell envelope [49]. Atypical among bacteria, the *M. tuberculosis* cell envelope has an elaborate dense multilayered structure devoid of the majority of transporters, with the wax-like outer membrane formed by mycolic acids and their derivatives [12,49]. Its penetration is believed to be facilitated by the relatively higher lipophilicity of anti-TB drugs (compared to other antibacterials), requiring reassessment of the standard drug-likeness rules [38,49,51] and the shift of the overall ADME analysis towards the local (microenvironment-based) drug exposure [52]. Although the whole-cell phenotypic screening followed by target elucidation is presently seen as the most efficient approach to the tuberculosis drug discovery [30,48], the ability to predict and optimize envelope permeability for a potential drug using in silico models would be very valuable in any pipeline.

The first steps towards this goal were made in the 1990s [53,54] by experimental permeability measurements in the model *Mycobacteria* species for limited sets of antibiotics and nutrients. They were shown to be much lower than in other bacteria, and rough semi-quantitative correlations with lipophilicity and charges were established, highlighting the diffusion-based and porin-assisted permeation mechanisms. In one study [55], the *M. tuberculosis* cell wall permeabilities for a small congeneric series of antitubercular drugs were estimated simply as their Caco-2 cell membrane permeabilities using correlations with several physico-chemical descriptors. In another study [56], based on the simplistic molecular dynamics simulations of solutes in pseudo-mycolic acid monolayers, the lateral and transverse diffusion coefficients were calculated and the qualitative correlation between the solute molecular shape and permeability was established.

Taking into account the difficulties of direct permeability measurement, later research mostly relied on general-purpose quantitative structure–activity/structure–property relationship (QSAR/QSPR) modeling techniques applied to more or less representative structure–permeability datasets, wherein the permeability estimates were derived from the publicly available activity data. In the MycPermCheck model [57] for permeability classification, the 3727 compounds from the CDD TB database [58] that were active in the cell-based inhibition assays were considered as permeable, whereas the “impermeable” examples were generated by a random sampling of drug-like compounds from the ZINC12 database [59]. Using five previously selected physico-chemical descriptors, a one-dimensional principal component model, and logistic regression, the model achieved a sensitivity of 67.2% at the specificity of 90% (or sensitivity of 72.2% at the specificity of 75%) on the validation set.

In one study [60], the permeability was estimated from the ChEMBL [61] data using the differences in activity between the cell-based and enzyme-based *M. tuberculosis* inhibition assays. For various subsets of 366 common compounds and additional 273 compounds highly potent in cell-based assays, the Partial Least Squares Regression (PLSR) models based on the subset of PaDEL [62] 1D and 2D descriptors were built and further translated to classification predictions, and the sensitivity of 70–95% and specificity of 8–45% were achieved for the validation set. Developing this approach, the recent work [63] used a ChEMBL-derived dataset of 1114 compounds, PaDEL descriptors, and a variety of machine learning methods to achieve the area under ROC curve (AUC) value of 0.81 for the validation set of 40 compounds.

Inspired by these encouraging results, the goal of the present work was to develop a predictive in silico model of *Mycobacterium tuberculosis* permeability based on the available Big Data from the cell-based and enzyme-based inhibitory activity assays that would be applicable to diverse drugs and drug-like compounds.

## 2. Results and Discussion

### 2.1. General Modeling Approach

For a broadly applicable predictive structure–permeability model, a key foundational element is a sufficiently diverse and representative dataset. Following the approach proposed and validated in the previous studies, the permeability data were derived from the differences in the inhibitory activity measurements between the target-based and cell-based assays, as reported in the publicly available Big Data sources (see Section 2.2).

For model construction, we decided to focus on the combination of artificial neural networks and the fragmental (substructural) descriptors representing the occurrence numbers of various substructures. Providing efficient tools for various QSPR and QSAR problems [64,65,66], this approach has been successfully employed to model the structure influence on various pharmacokinetic, toxicity, and physico-chemical endpoints such as human intestinal absorption [67], blood–brain barrier permeability [68,69], hERG-mediated cardiac toxicity [70], lipophilicity [71], etc. Some of these models are available online at our ADMET Prediction Service page (http://qsar.chem.msu.ru/admet/ accessed on 1 December 2022) and have been successfully used to evaluate the key absorption, distribution, metabolism, excretion, and toxicity (ADMET) properties of diverse potential drug compounds in virtual screening and molecular design studies [72,73,74,75,76].

### 2.2. Mycobacterium tuberculosis Inhibitor Permeability Dataset

As noted above, similar to the previous studies [60,63], the compounds that have shown activity in any of the selected target-based assays were classified as permeable if they were active in any of the selected cell-based assays; otherwise they were taken to be impermeable. To this end, the publicly available PubChem 2022 database [77] was used as the source of (Big) raw data (in particular, it included both the assays synchronized with ChEMBL [61] and a number of additional assays). The Big Data resources offer unprecedented opportunities for deep analysis of the structure–activity and structure–property relationships and for the development of more accurate and broadly applicable predictive models, but require additional efforts for data preparation and curation [78]. On the other hand, one should bear in mind that large diverse datasets, often comprising compounds with different properties and mechanisms of action and based on only partially comparable measurements (commonly approximate by design) that are performed in different laboratories over significant time periods using varying techniques and conditions, usually impose natural limits on the quality of the resulting models.

The detailed data preparation procedures reflecting the established guidelines [79,80,81] are explained in Section 3.1, and only a brief overview of salient points is presented here. Using the automated keyword search in the local database, the assays potentially relevant for the antimycobacterial activity were identified. During the expert analysis of this list, the key cell-based and target-based assays were selected that were required to be sufficiently populated in terms of data point counts as well as sufficiently diverse and representative in the chemical and endpoint spaces. In particular, the chemical space coverage was prioritized over the maximum reliability of specific data points, and this was reflected both in the assay selection and in the downstream standardization of the (binary) activity definitions.

Using custom Web scripts accessing both local and remote databases, the assay and compound data were joined and downloaded as the TSV format files. After manual and automated preprocessing and curation, the cleaned individual datasets were prepared and then merged to produce the united datasets for the selected cell-based and target-based assays. In total, the cell-based dataset contained 557,527 compounds, among which 96,040 compounds were active in at least one out of 11 assays. The target-based dataset contained 926,660 compounds, among which 9450 compounds were active in at least one out of 11 assays.

By matching this target-active dataset against the cell-based results, 8242 compounds were identified that were active in at least one target-based assay and have also been tested in at least one cell-based assay. In particular, 2671 compounds that have shown activity in at least one cell-based assay were classified as penetrating the *M. tuberculosis* cell wall (*MtbPen* = 1, positive result) whereas the remaining 5571 compounds (inactive in all 11 cell-based assays) were classified as non-penetrating (*MtbPen* = 0, negative result). In the present paper, this dataset will be called *MtbPen8242*.

As can be seen, the *MtbPen8242* dataset is moderately imbalanced (the ratio of non-penetrating to penetrating compounds is greater than 2.08). During subsequent structure-property modeling, this imbalance was found to create problems limiting the model quality. Thus, a balanced dataset *MtbPen5371ad* of 5371 compounds was prepared from it that comprises all (2671) penetrating compounds as well as a diverse subset of 2700 out of 5571 non-penetrating compounds. This dataset is provided in the Supplementary Materials.

### 2.3. Molecular Descriptors

The fragmental (substructural) descriptors [64,65,66] representing the occurrence numbers of various substructures were calculated in the framework of the NASAWIN 2.0 [82] software. Linear paths, cycles, and branches were generated using multi-level classification that takes into account atom types, valence states, bonding patterns, and number of attached hydrogens as well as bond types. The rare fragments that are present in fewer than 100 compounds and thus cannot be used to detect general predictive relationships were removed. The fragments containing up to eight non-hydrogen atoms were considered in order to provide sufficiently detailed description of the structures without excessive increase in the number of descriptors. In total, several thousands of descriptors (depending on the fragment size) were generated.

### 2.4. Neural Network Modeling Procedure

As noted above, similar to our studies on the prediction of ADMET properties [67,68,69,70], the combination of fragmental descriptors and artificial neural networks is especially suitable for modeling such primarily non-specific properties in diverse sets of organic and drug-like compounds. Even the specific contributions (e.g., from various active transporters) are implicitly taken into account by the neural network-based fragmental model [69].

**Figure 1:**
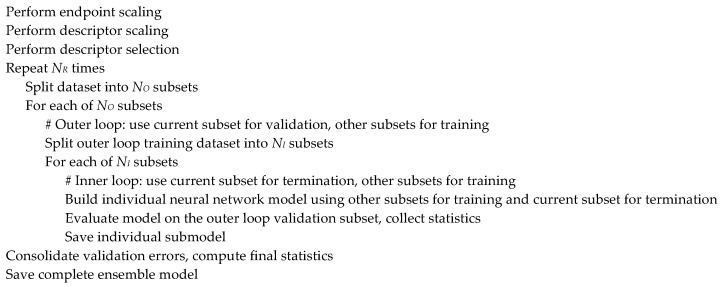
General modeling workflow.

Further developing the previously published modeling approach [69], we created a novel network architecture that logically implements the same high-level modeling workflow, integrating the classical feed-forward back-propagation neural network (BPNN) and the repeated double cross-validation [83] approach (Figure 1). The double cross-validation procedure involves two loops, and in each loop a fraction of the dataset is randomly selected as a test subset. During each iteration of the inner loop, a neural network submodel is built using the training subset while the prediction error on the test subset is monitored to provide the early termination while the outer loop test subset is used to validate the resulting model. Usually, the 5 × 4-fold double cross-validation scheme is employed, corresponding to $N_{O}=5$ and $N_{I}=4$ in Figure 1. That is, in the outer loop, the dataset is split into five subsets of approximately equal sizes and each of them is used to validate four models built in the inner loop by splitting the remaining data into four subsets of approximately the same size and using three of them for training the model and one for early termination. The procedure can be repeated several times ($N_{R}$) to enhance the stability and reliability of the results [69]. The validation subset errors are then consolidated and normalized into the appropriate cross-validation statistics (such as the accuracy, balanced accuracy, sensitivity, and specificity for classification models). To reduce the risk of overfitting and chance correlations, the inner and outer splits are randomly shuffled at each step. This approach not only provides quite reliable estimates of the model predictivity but also generates an ensemble of neural network models based on different subsets of data that can be used to improve prediction quality and evaluate the model applicability.

**Figure 2:**
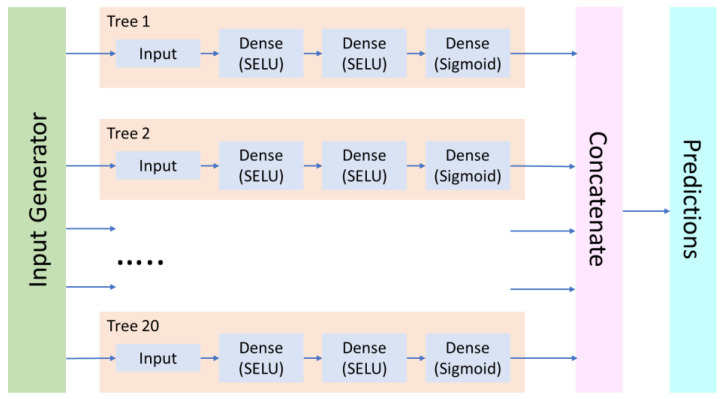
Basic architecture of the “forest” neural network model.

However, although most commonly the double cross-validation procedure is performed sequentially, in the present work, we implemented a parallelized version that unrolls the loops and integrates the ensemble submodels (“trees”) into a single neural network (“forest”) that is fed with input data from the generator objects. This approach significantly enhanced the modeling performance (Figure 2).

Most of the other key architecture decisions from the earlier study [69] were retained (see the reference for the discussion of other available options). Each “tree” neural subnetwork may include one or more fully connected (*Dense*) layers with the scaled exponential linear unit (SELU) activation function [84] that provides the best results in terms of model quality and training efficiency. Optionally, the fully connected layers can be interleaved with the *AlphaDropout* [84] regularization layers in order to prevent overfitting. For the output layer in a classification model, the *sigmoid* activation function was used, which is expected to provide an estimated probability of the compound penetrating the cell wall (positive result). Binary cross-entropy (BCE) was used as a loss function for model training. For data preprocessing, the *MinMaxScaler* algorithm was used, which transforms the descriptors by linear scaling to the [0, 1] range. Global descriptor selection (for the entire modeling dataset after scaling) was performed to remove low-variable descriptors (defined as variance below 10^–6^) and to identify the most relevant descriptor subset using a stepwise descriptor selection procedure wherein the Partial Least Squares regression model is iteratively refined by adding descriptors with the highest F-value score with the residual endpoint. Since these models are sufficiently different from the resulting neural network models, we can be reasonably confident that the descriptor selection procedure does not lead to overfitting or chance correlations.

The neural network models were built using the in-house Python script based on the TensorFlow 2.4.1/Keras 2.4.3 framework on a high-performance NVIDIA RTX3080Ti GPU.

The hyperparameters controlling the machine learning modeling workflow can significantly affect its quality and efficiency. These include the neural network architecture (number and size of the hidden layers) and training parameters as well as the descriptor set (in particular, fragment size, selection algorithm, and the number of selected descriptors) and the prediction and applicability control parameters (see below). In the present study, hyperparameter optimization and model selection were performed using the Optuna 3.0.3 [85] library that implements the tree-structured Parzen Estimator algorithm. The goal function for the maximization was defined as the cross-validated balanced accuracy of the model. For some of the hyperparameters, the optimal values determined in the preliminary tests were kept fixed during the final modeling.

As mentioned above, an ensemble of the neural network submodels generated by the double cross-validation procedure from different subsets of data can be used to improve prediction quality and evaluate the model applicability. In particular, for the classification case, the mean and standard deviation of the individual predicted probability values are computed, and a failed prediction is reported if the standard deviation is greater than a specified fraction of the acceptable range (usually 30%).

### 2.5. Predictive Model of Mycobacterium tuberculosis Permeability

For the full *MtbPen8242* dataset, three sets of fragmental descriptors were considered during the hyperparameter optimization, containing up to 5, 6, or 8 non-hydrogen atoms. Descriptor subsets of varying size (from 100 to 1000 descriptors) were selected. Two or three hidden layers were considered in the neural network whereas the size of hidden layers relative to the number of descriptors was varied in the ranges 0.80–0.01 and 0.30–0.10 or 0.80–0.01, 0.50–0.01, and 0.30–0.10, respectively. The dropout layers with probability between 0 and 0.5 were used. The optimal model was based on 500 fragmental descriptors containing up to six non-hydrogen atoms, and two hidden layers containing 296 and 75 neurons. Unfortunately, its predictivity was lower than desired (cross-validated accuracy *Acc_cv_* = 0.752, balanced accuracy *BalAcc_cv_* = 0.683, sensitivity *Sens_cv_* = 0.486, and specificity *Spec_cv_* = 0.880, the confusion matrix is presented in Table 1). These data, as well as the inspection of individual predictions, indicated that the model failed to recognize many of the penetrating compounds, producing many false negatives. It was suggested that this bias could be caused by the dataset imbalance, with excessive non-penetrating compounds implicitly increasing their importance and the model’s preference for them.

**Figure 3:**
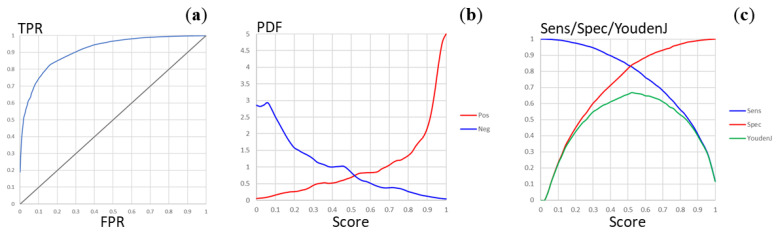
Plots of the model quality parameters for the *MtbPen* classification model. (**a**) The ROC curve (TPR, true positive rate; FPR, false positive rate). (**b**) The distribution densities (PDF, probability density function) of the penetration probability scores for the positive and negative compounds. (**c**) Dependence of the sensitivity, specificity, and Youden’s *J* statistic on the score threshold.

For this reason, the balanced *MtbPen5371ad* dataset was prepared as described in Section 2.2, and the new model was built using hyperparameter optimization with the same search space definition, except that the fragmental descriptors up to eight atoms were not considered. The optimal model was based on 900 fragmental descriptors containing up to six non-hydrogen atoms, and two hidden layers containing 46 and 270 neurons (interestingly, the network architectures with three hidden fully connected layers did not provide significant improvements in model quality). The predictivity of this model was significantly higher, with cross-validated accuracy *Acc_cv_* = 0.768, balanced accuracy *BalAcc_cv_* = 0.768, sensitivity *Sens_cv_* = 0.768, and specificity *Spec_cv_* = 0.769 (the confusion matrix is presented in Table 1). The ROC curve for this classification is shown in Figure 3A. The area under ROC curve can be calculated as *AUCROC* = 0.911. The plot of the distribution densities of probability scores for the positive and negative compounds (Figure 3B) demonstrates good separation of the penetrating and the non-penetrating compounds whereas the plots of the sensitivity, specificity, and Youden’s *J* statistic values *vs* the score threshold (Figure 3C) show that the natural threshold of 0.5 is close to optimal. These parameters are similar or better than those of the most reliable models available in the literature, whereas a substantially broader applicability domain can be expected thanks to the significantly larger, representative, and diverse training set. The training of the model was completed in about 260 epochs, indicating low risk of overfitting. Nevertheless, one should bear in mind that the uncertainty of the data (stemming from the trade-offs inherent in high-throughput screening as well as from certain heuristics employed in the analysis) could limit the model predictivity.

Overall, the resulting predictive model can provide useful guidance and improve the efficiency of the virtual screening, multiparameter assessment, and lead optimization efforts for the potential antitubercular drugs. However, similar to any in silico model, its predictions should eventually be validated in vitro and/or in vivo since a specific compound of interest might be outside of the model applicability domain or could interact with the *M. tuberculosis* cell wall components (such as transporters) in some unexpected ways.

## 3. Materials and Methods

### 3.1. Mycobacterium tuberculosis Inhibitor Permeability Dataset

As noted above, similar to the previous studies [60,63], the compounds that have shown activity in any of the selected target-based assays were classified as permeable if they were active in any of the selected cell-based assays, otherwise, they were taken to be impermeable. As the source of (Big) raw data, the publicly available PubChem 2022 database [77] was employed. Using the automated keyword search in the local database (assay name or description contain “mycobacter” or “tubercul”), the assays potentially relevant for the antimycobacterial activity were identified. During the expert analysis of this list, the key cell-based and target-based assays for *Mycobacterium tuberculosis* inhibition were selected that were required to be sufficiently populated in terms of data point counts as well as sufficiently diverse and representative in the chemical and endpoint spaces (Table 2). In particular, the chemical space coverage was prioritized over the maximum reliability of specific data points, and this was reflected both in the assay selection and in the downstream standardization of the (binary) activity definitions that was based preferentially on the primary screening inhibition percentages rather than on more accurate but much less abundant secondary screening data.

DataWarrior 5.5.0 software (Idorsia Pharmaceuticals Ltd., https://openmolecules.org/ accessed on 1 December 2022) was used for the management, search, and analysis of the structure–activity databases.

Using custom PHP Web scripts accessing both local and remote databases, the assay and compound data were joined and downloaded as the TSV format files. The cleaned individual datasets were prepared using manual and automated data preprocessing and curation involving the removal of unnecessary data columns, deduplication of activity records for the compounds (that could contain different activities or equivalent or different results of repeated measurements of the same activity), standardization of the chemical structures (removal of smaller disconnected fragments, neutralization of salts), and standardization of the (binary) activity definitions and representations (see Table 2). Then, the individual datasets were merged to produce the united datasets for the selected cell-based and target-based assays. In total, the cell-based dataset contained 557,527 compounds, among which 96,040 compounds were active in at least one out of 11 assays. The target-based dataset contained 926,660 compounds, among which 9450 compounds were active in at least one out of 11 assays.

By matching this target-active dataset against the cell-based results, 8242 compounds were identified that were active in at least one target-based assay and have also been tested in at least one cell-based assay. In particular, 2671 compounds that have shown activity in at least one cell-based assay were classified as penetrating the *M. tuberculosis* cell wall (*MtbPen* = 1, positive result) whereas the remaining 5571 compounds (inactive in all 11 cell-based assays) were classified as non-penetrating (*MtbPen* = 0, negative result). In the present paper, this dataset is called *MtbPen8242*.

Since the *MtbPen8242* dataset is moderately imbalanced (the ratio of non-penetrating to penetrating compounds is greater than 2.08), a balanced dataset *MtbPen5371ad* was prepared from it that comprises all (2671) penetrating compounds as well a diverse subset of 2700 out of 5571 non-penetrating compounds. This dataset is provided in the Supplementary Materials.

### 3.2. Modeling Workflow

The fragmental (substructural) descriptors representing the occurrence numbers of various substructures were calculated in the framework of the NASAWIN 2.0 [82] software. Linear paths, cycles, and branches were generated using multi-level classification that takes into account atom types, valence states, bonding patterns, and number of attached hydrogens as well as bond types. The rare fragments that are present in fewer than 100 compounds and thus cannot be used to detect general predictive relationships were removed. The fragments containing up to 8 non-hydrogen atoms were considered.

Predictive neural network models were built using the in-house Python script based on the TensorFlow 2.4.1/Keras 2.4.3 framework on a high-performance NVIDIA RTX3080Ti GPU. In addition to the standard libraries, the *scikit-learn* 1.1.3 machine learning framework [86] and the Optuna 3.0.3 [85] hyperparameter optimization library were used.

## 4. Conclusions

Thus, we have developed a predictive in silico model of the *Mycobacterium tuberculosis* cell wall permeability (*MtbPen*) derived from the extensive Big Data-based dataset and applicable to diverse drugs and drug-like compounds. Using the fragmental (substructural) descriptors representing the occurrence numbers of various substructures, we have refined the modeling workflow and evaluated the performance of different options. Playing a key role, the double cross-validation procedure generates an ensemble of neural network models based on different subsets of data that can be used to improve prediction quality and to evaluate the model applicability for a particular compound. Its novel parallelized implementation integrates the ensemble submodels (“trees”) into a single neural network (“forest”) that is fed with input data from the generator objects. This approach significantly enhanced the modeling performance. It was also found that even moderate (2:1) dataset imbalance could degrade the model quality since the excessive non-penetrating compounds implicitly increase their importance and the model’s preference for them.

Our optimal model is based on a balanced dataset of 5371 compounds (including 2671 penetrating compounds as well as a diverse representative subset of 2700 non-penetrating compounds) and 900 fragmental descriptors of up to six non-hydrogen atoms. It has quite good predictivity parameters (cross-validated accuracy *Acc_cv_* = 0.768, balanced accuracy *BalAcc_cv_* = 0.768, sensitivity *Sens_cv_* = 0.768, specificity *Spec_cv_* = 0.769, and area under ROC curve *AUCROC* = 0.911) that are similar or better than those of the most reliable models available in the literature, whereas a substantially broader applicability domain can be expected thanks to the significantly larger, representative, and diverse training set. The model can provide useful guidance and improve the efficiency of the virtual screening, multiparameter assessment, and lead optimization efforts for potential antitubercular drugs. However, similar to any in silico model, its predictions should eventually be validated in vitro and/or in vivo since a specific compound of interest might be outside of the model applicability domain or could interact with the *M. tuberculosis* cell wall components (such as transporters) in some unexpected ways.

This predictive model will be made available online at our ADMET Prediction Service page (http://qsar.chem.msu.ru/admet/ accessed on 1 December 2022), enabling the evaluation and optimization of the *Mycobacterium tuberculosis* cell wall permeability and other key ADMET properties of potential antitubercular agents and other drug compounds.

## Figures and Tables

**Table 1:** Confusion matrices for the *MtbPen* classification models.

Dataset		MtbPen8242		MtbPen5371ad	
		**Predicted**		**Predicted**	
		**Positive**	**Negative**	**Positive**	**Negative**
**Observed**	**Positive**	1435	1236	2214	457
	**Negative**	528	5043	437	2263

**Table 2:** PubChem assays used in compiling the *MtbPen* datasets.

AID ^1^	ID	Type	Activity/Compound Count ^2^	Description	Activity Condition ^3^
1332	C01	Cell	1118	High throughput screen to identify inhibitors of *Mycobacterium tuberculosis* H37Rv	Inh30
1626	C02	Cell	215,397	High throughput screen to identify inhibitors of *Mycobacterium tuberculosis* H37Rv	Inh30
1949	C03	Cell	100,697	High throughput screen of 100,000 compound library to identify inhibitors of *Mycobacterium tuberculosis* H37Rv	Inh30
2842	C04	Cell	23,823	High throughput screen of a putative kinase compound library to identify inhibitors of *Mycobacterium tuberculosis* H37Rv	Inh30
449762	C05	Cell	327,669	High throughput screening assay used to identify novel compounds that inhibit *Mycobacterium tuberculosis* in 7H9 media	Inh30
1259343	C06	Cell	6225	High throughput screening of small molecules that kill *Mycobacterium tuberculosis*	Inh30
1259417	C07	Cell	1105	High throughput whole cell screen to identify inhibitors of *Mycobacterium tuberculosis*	Inh30
1671161	C08	Cell	96,022/86,588	Phenotypic growth assay for *Mycobacterium tuberculosis* grown for 4 days on DPPC, cholesterol, tyloxapol-based media	Inh30
1671162	C09	Cell	103,984/86,574	Phenotypic growth assay for *Mycobacterium tuberculosis* grown for 3 days on 7H9, glucose tyloxapol-based media	Inh30
1671174	C10	Cell	53,171/53,165	Phenotypic assay to identify agents that inhibit growth of *Mycobacterium tuberculosis*	Inh30
488890	C11	Cell	324,545	Elucidation of physiology of non-replicating, drug-tolerant *Mycobacterium tuberculosis*	Inh30
375	T01	Target	10,011/10,009	*Mycobacterium tuberculosis* pantothenate synthetase assay	Outcome
1376	T02	Target	216,162/215,860	Inhibitors of mycobacterial glucosamine-1-phosphate acetyl transferase (GlmU)	Outcome
2606	T03	Target	324,858/324,747	Primary biochemical high throughput screening assay to identify inhibitors of the membrane-associated serine protease Rv3671c in *M. tuberculosis*	Outcome
504406	T04	Target	324,148/324,048	High throughput screening of inhibitors of *Mycobacterium tuberculosis* UDP-galactopyranose mutase (UGM) enzyme	Outcome
540299	T05	Target	103,205/102,628	A screen for compounds that inhibit the MenB enzyme of *Mycobacterium tuberculosis*	Outcome
588335	T06	Target	356,407/356,160	Counterscreen for inhibitors of the fructose-bisphosphate aldolase (FBA) of *M. tuberculosis*	Outcome
602481	T07	Target	356,486/353,572	*Mycobacterium tuberculosis* BioA enzyme inhibitor	Outcome
1159583	T08	Target	301,203/300,060	High throughput screen for small molecule inhibitors of a hypoxia-regulated fluorescent biosensor in *Mycobacterium tuberculosis*	Outcome
1671160	T09	Target	8874/8841	Assay for Asp RNA synthetase-1 from *Mycobacterium tuberculosis*	Inh30
1671178	T10	Target	67,199/66,591	*Mycobacterium tuberculosis* polyketide synthase 13 thioesterase (PKS13)	Inh30
2221	T11	Target	293,466/293,376	Cell-free homogenous primary high throughput screen to identify inhibitors of RecA intein splicing activity	Outcome

^1^ PubChem assay ID. ^2^ Number of raw activity records and (if different) number of compounds after deduplication and preprocessing. ^3^ Conditions used to identify active compounds: Inh30–Inhibition > 30%; Outcome–Activity Outcome = Active.

## Data Availability

The data presented in this study are available on request from the corresponding author.

## Supplementary Materials

The following supporting information can be downloaded at: https://www.mdpi.com/article/10.3390/molecules28020633/s1, structure–permeability dataset.
